# Potassium Diformate Attenuates Inflammation and Enhances Intestinal Barrier Function Associated With Gut Microbiota Modulation in Nursery Piglets

**DOI:** 10.1002/fsn3.71888

**Published:** 2026-06-24

**Authors:** Tiande Zou, Lina Zhang, Qin Li, Yong Cheng, Li Lu, Songtao Zhong, Mingren Qu, Jun Chen

**Affiliations:** ^1^ Jiangxi Province Key Laboratory of Animal Nutrition and Feed, Jiangxi Province Key Innovation Center of Integration in Production and Education for High‐Quality and Safe Livestock and Poultry Jiangxi Agricultural University Nanchang China

**Keywords:** gut microbiota, inflammation, intestinal barrier function, nursery piglets, potassium diformate

## Abstract

This experiment explored the impacts of potassium diformate (K‐diformate) on growth performance, inflammation, intestinal barrier function, and gut microbiota in nursery piglets. Twenty‐four weaned piglets were assigned to four dietary groups supplemented with 0%, 0.6%, 1.2%, or 1.8% K‐diformate for 28 days. Results showed that the FCR decreased linearly with increasing K‐diformate levels (*p* < 0.05). Importantly, piglets in the 1.8% K‐diformate group had the lowest FCR, which was lower than that of the control group by 15.03% and 14.29% in weeks 1–2 and 3–4, respectively (*p* < 0.05). Additionally, piglets receiving 1.8% K‐diformate demonstrated lower serum IL‐6 levels (9.27% reduction), along with reduced jejunal IL‐6 (17.64%), IL‐1β (10.29%), and TNF‐α levels (14.01%) relative to the control group (*p* < 0.05). Furthermore, piglets in the 1.8% K‐diformate group exhibited decreased serum D‐lactate (36.00% reduction) and LPS levels (9.90% reduction), and elevated serum GLP‐2 levels (9.23% increase), as well as upregulated jejunal ZO‐1, Occludin, and MUC2, both at mRNA and protein expression levels (*p* < 0.05). Microbiota analysis revealed piglets in the 1.8% K‐diformate group exhibited higher abundances of *Treponema*, *Roseburia*, *Rikenellaceae_RC9_gut_group*, and *norank_f_Eubacterium_coprostanoligenes_group*, while showing lower abundances of *Lactobacillus*, *Streptococcus*, *Sarcina*, and *Blautia* (*p* < 0.05). Correlation analysis further revealed associations between *Roseburia*, *Blautia*, *Sarcina*, and *Lactobacillus* with inflammation, and between *Treponema*, *Roseburia*, *Streptococcus*, *Blautia*, and *Sarcina* with intestinal barrier function (*p* < 0.05). Collectively, dietary addition with K‐diformate (especially 1.8%) improved growth performance, inflammation, and intestinal barrier function associated with gut microbiota modulation in nursery piglets.

## Graphical abstract

Twenty‐four weaned piglets were assigned to four dietary groups supplemented with 0%, 0.6%, 1.2%, or 1.8% K‐diformate for 28 days. Dietary addition with K‐diformate (especially 1.8%) improved growth performance, inflammation, and intestinal barrier function associated with gut microbiota modulation in nursery piglets.

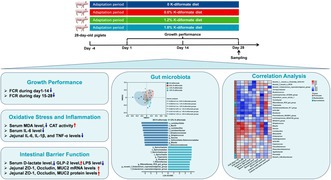

## 1 Introduction

The nursery phase represents a pivotal stage in swine production (Barbosa et al. 2022), wherein weaned piglets face multiple challenges, including weaning stress, environmental changes, and pathogenic infections, all of which adversely affect the gut microbiota (Upadhaya and Kim 2022). The intestinal microbial ecosystem is fundamental in maintaining optimal physiological, nutritional, and immune functions in swine (Fouhse et al. 2016). A disrupted gut ecosystem can significantly reduce functional richness and microbial diversity, impair metabolic functions (Upadhaya and Kim 2022), and often lead to inflammation (Bin et al. 2018) and compromised intestinal barrier function (Di Vincenzo et al. 2024). Therefore, dietary interventions to modulate intestinal microbiota, mitigate inflammation, and improve intestinal barrier function in weaned piglets have gained increasing attention in animal nutrition research.

Potassium diformate (K‐diformate), a double‐salt form of formic acid (HCOOH·HCOOK), has emerged as a promising feed additive with demonstrated antimicrobial and gut‐modulating properties (Chen, He, et al. 2024; Chen, Zheng, et al. 2024; Sun et al. 2022). K‐diformate consists of alternating potassium ion and formate dimers connected by ionic interactions (Larsson and Nahringbauer 1968). Compared to formic acid, potassium formate exhibits greater stability, with a melting point of 108.6°C. It does not volatilize at room temperature and lacks a pronounced irritating odor. As such, in contrast to formic acid, K‐diformate exhibits enhanced stability in feed and reduced corrosivity, rendering it more suitable for practical application in animal nutrition. It provides a safer and more stable approach for maintaining the equilibrium of microbial populations in animals (Chen, He, et al. 2024). For instance, dietary inclusion with 0.4% or 0.8% K‐diformate for 42 days markedly reduced the counts of *Escherichia coli* , *Lactobacilli*, *Bifidobacteria*, and *Bacillus* in the ileum of broilers (Chen, Zheng, et al. 2024). Similarly, dietary supplementation with 1.2% K‐diformate for 42 days increased the abundances of Firmicutes, *Clostridium_sensu_stricto_1*, and *Unclassified_Erysipelotrichaceae*, while reducing Fusobacteriota abundance in the cecal microbiota of Cherry Valley ducks (Li et al. 2024). Additionally, our previous studies have demonstrated that organic acids can improve intestinal barrier function in animals (Chen, Chen, et al. 2024; Chen et al. 2023, 2025). However, to date, only one in vitro study using porcine intestinal epithelial cells has investigated the effects of K‐diformate on tight junction proteins. Lin et al. (2024) found that K‐diformate positively influenced tight junction proteins, as evidenced by upregulated Occludin expression in IPEC‐J2 cells infected with enterohemorrhagic *Escherichia coli* . These findings suggest that K‐diformate has potential for enhancing intestinal barrier function in nursery piglets. However, the effects of K‐diformate on gut microbiota diversity, composition, microbial metabolites, and intestinal barrier function in nursery piglets remain incompletely understood.

Therefore, this experiment was performed to explore the impacts of dietary supplementation with varying levels of K‐diformate on inflammation response, intestinal barrier function, gut microbiota, and microbial metabolites in nursery piglets. Furthermore, correlation analysis was conducted to explore the potential relationships between microbial alterations and host serum inflammation as well as serum intestinal barrier biomarkers, thereby providing a theoretical basis for the rational use of K‐diformate in piglet nutrition.

## 2 Materials and Methods

### 2.1 Experimental Design

Twenty‐four castrated DLY male piglets, originating from six litters (four piglets per litter) and weaned at 28 days of age, were selected in this experiment. The pigs were clinically healthy with no signs of diarrhea and had a mean body weight of 8.0 kg. After 4‐day acclimation, the piglets were randomly allotted into four groups based on initial body weight and litter, with six replicates per group and one pig per replicate. To ensure litter balance, piglets from each sow were evenly distributed across the four experimental groups. The piglets in the control group received a basal diet, while the piglets in the remaining three groups were administered the basal diet added with 0.6%, 1.2%, and 1.8% K‐diformate (98% purity), respectively. K‐diformate was incorporated into the basal diet by replacing equivalent quantities of zeolite in the premix of the three K‐diformate‐supplemented diets. The basal diet was prepared to meet the nutrient requirements of piglets according to the National Research Council, Division on Earth, Life Studies, and Committee on Nutrient Requirements of Swine (2012). The composition of ingredients and nutritional content of the basal diet are detailed in Table 1. The experiment was conducted over a 28‐day period, with piglets provided unrestricted access to feed and water throughout the duration. During the experiment, the piglets remained clinically healthy without obvious signs of diarrhea or anorexia.

**TABLE 1:** Ingredient composition and nutritional levels of the basal diet.

Ingredient	Content (%)	Nutrient level	Content
Expanded corn	35.00	Digestible energy, MJ/kg^b^	14.79
Corn	27.78	Organic matter, %^c^	83.57
Extruded soybean	10.00	Crude protein, %^c^	17.71
Soybean meal	8.00	Calcium, %^c^	0.81
Soybean protein concentrate	5.00	Total phosphorus, %^c^	0.62
Whey powder	4.00	Available phosphorus, %^b^	0.45
Soybean oil	3.00		
Fish meal	2.00		
Premix^a^	5.22		
Total	100.00		

^a^ The premix provides nutritional composition per kilogram of complete diet: vitamin A (6000 IU), vitamin D_3_ (400 IU), vitamin E (10 IU), vitamin K_3_ (2 mg), vitamin B_1_ (0.8 mg), vitamin B_6_ (2.4 mg), vitamin B_12_ (0.012 mg), folic acid (0.2 mg), niacin (14 mg), pantothenic acid (10 mg), zinc (80 mg), manganese (40 mg), iron (100 mg), copper (10 mg), iodine (0.3 mg), and selenium (0.3 mg).

^b^ Calculated values.

^c^ Analyzed values.

### 2.2 Data and Sample Collection

The body weights of the piglets were measured at the commencement of the trial (day 1), as well as on days 14 and 28, while their feed consumption was systematically documented. Utilizing these measurements, the average daily gain (ADG), average daily feed intake (ADFI), and feed conversion ratio (FCR) were subsequently computed. On day 28, blood was sampled from the anterior vena cava, and serum was harvested via centrifugation at 3000 r/min for 15 min (Wang et al. 2023), followed by storage at −80°C. After blood sampling, the piglets were euthanized in accordance with ethical guidelines, after which samples of mid‐jejunal mucosa and colonic digesta were collected and preserved at −80°C.

### 2.3 Sample Analysis

#### 2.3.1 Measurement of Oxidative Stress and Inflammatory Parameters

The malondialdehyde (MDA) level, catalase (CAT) activity, total superoxide dismutase (T‐SOD) activity, total antioxidant capacity (T‐AOC), diamine oxidase (DAO) activity, and glucagon‐like peptide‐2 (GLP‐2) level in the serum were quantified with commercial kits (Jiancheng, Nanjing, China). The jejunal mucosa was homogenized in saline solution (1:10, w/v), and the supernatants were analyzed for protein concentration using a BCA kit (Jiancheng, Nanjing, China). The supernatants were then adjusted with saline solution to ensure uniform protein concentration across all samples. Subsequently, the adjusted supernatants were analyzed for interleukin‐6 (IL‐6), interleukin‐1β (IL‐1β), and tumor necrosis factor‐α (TNF‐α) levels using ELISA kits (Shanghai Enzyme‐Linked Biotechnology, Shanghai, China). Serum levels of IL‐6, IL‐1β, TNF‐α, D‐lactate, and lipopolysaccharide (LPS) were determined using ELISA kits (Shanghai Enzyme‐Linked Biotechnology, Shanghai, China).

#### 2.3.2 RT‐qPCR Analysis

The RT‐qPCR analysis was performed as described in our previous study (Chen et al. 2026). Briefly, total RNA was extracted from jejunal mucosal samples using the RNA extraction kit (Accurate, Changsha, China). RNA concentration was measured using an ultramicro nucleic acid protein analyzer (BioDrop, Cambridge, UK), and RNA quality was assessed by agarose gel electrophoresis. All samples were adjusted to a concentration of 500 ng/μL. And then, cDNA synthesis was performed using a reverse transcription kit (Accurate, Changsha, China). Quantitative real‐time PCR was carried out on a CFX96 RT‐PCR detection system (Bio‐Rad, Hercules, CA, USA) with TransStart Top Green qPCR SuperMix (Accurate, Changsha, China). β‐actin was used as the internal reference gene, and target gene mRNA expression levels were calculated using the 2^−ΔΔ*CT*^ method (Livak and Schmittgen 2001). Primer sequences are provided in Table 2.

**TABLE 2:** The primers used for RT‐qPCR.

Gene	Accession number	Primer sequences (5′‐3′)
*ZO‐1*	XM_013993151.1	F: CTGAGGGAATTGGGCAGGAA R: TCACCAAAGGACTCAGCAGG
*MUC2*	XM_013989745.1	F: GGTCATGCTGGAGCTGGACAGT R: TGCCTCCTCGGGGTCGTCAC
*Occludin*	NM_001163647.2	F: CAGGTGCACCCTCCAGATTG R: GGACTTTCAAGAGGCCTGGAT
*β‐actin*	DQ178122	F: TCTGGCACCACACCTTCT R:TGATCTGGGTCATCTTCTCAC

#### 2.3.3 Western Blot Analysis

Western blot analysis was done as previously described (Zhong et al. 2024). Total protein was extracted and determined using a BCA assay kit (Jiancheng, Nanjing, China). Proteins were separated on a 10% SDS‐PAGE gel and transferred to a PVDF membrane. The membrane was blocked with 5% skimmed milk and incubated overnight at 4°C with primary antibodies under gentle agitation, followed by incubation with a secondary antibody for 2 h. Primary antibodies used were as follows: ZO‐1 (1:1500, Proteintech, Wuhan, China), Occludin (1:1500, Proteintech, Wuhan, China), and MUC2 (1:1500, Proteintech, Wuhan, China). Protein bands were visualized and quantified using ImageJ 1.53t software. β‐actin served as the internal reference for normalization of target protein expression levels.

#### 2.3.4 Gut Microbiota Analysis

Genomic DNA was extracted using a DNA extraction kit (Omega Bio‐tek, Norcross, GA, USA). The quality and concentration of the genomic DNA were assessed using 1% agarose gel electrophoresis and NanoDrop 2000 spectrophotometer, respectively. The amplification of bacterial 16S rRNA genes was performed employing universal bacterial primers 27F (5′‐AGRGTTYGATYMTGGCTCAG‐3′) and 1492R (5′‐RGYTACCTTGTTACGACTT‐3′). The PCR products were purified andsequenced using the Illumina Nextseq2000 platform (Illumina, San Diego, USA). Bioinformatic analysis was conducted using the Majorbio Cloud platform. Briefly, α‐diversity indices were calculated using Mothur v1.30.1. β‐Diversity was assessed, and statistical significance among treatments was determined using Adonis analysis with the Vegan v2.5‐3 package. LEfSe analysis was applied to identify significantly abundant bacterial taxa (phylum to genus) across different treatment groups (LDA score > 2, *p* < 0.05). The predicted functions of microbiota were analyzed using PICRUSt2. Spearman correlation analysis of microbiota at the genus level with FCR and serum parameters was also conducted.

#### 2.3.5 Analysis of Short‐Chain Fatty Acids

Short‐chain fatty acids were measured as outlined in our previous study (Chen et al. 2025). Briefly, 1.0 g of digesta was weighed, and distilled water was added at a volume‐to‐weight ratio of 1:5. The mixture was incubated at 4°C for 1 h and centrifuged at 12,000 rpm for 15 min. A 0.9 mL aliquot of the supernatant was mixed with 0.1 mL of 25% metaphosphoric acid solution, thoroughly vortexed, and incubated at 4°C for 1 h. After centrifugation at 15,000 rpm for 15 min, the supernatant was filtered. The filtrate was measured using a gas chromatographic system (Agilent, Santa Clara, CA, USA) to quantify short‐chain fatty acids.

### 2.4 Data Analysis

The requirement for statistical power was met by a sample size of six according to prior piglet studies (Chang et al. 2023; Sun et al. 2025; Zeebone et al. 2023). All data, except for gut microbiota, were processed using SPSS 25.0 software (IBM, Chicago, IL, USA). Orthogonal polynomial contrasts were conducted for linear effects, followed by one‐way ANOVA and Tukey's HSD post hoc test. A *p*‐value below 0.05 was considered statistically significant, while a *p*‐value ranging from 0.05 to 0.10 was regarded as indicative of a statistical trend.

## 3 Results

### 3.1 Growth Performance

**FIGURE 1:**
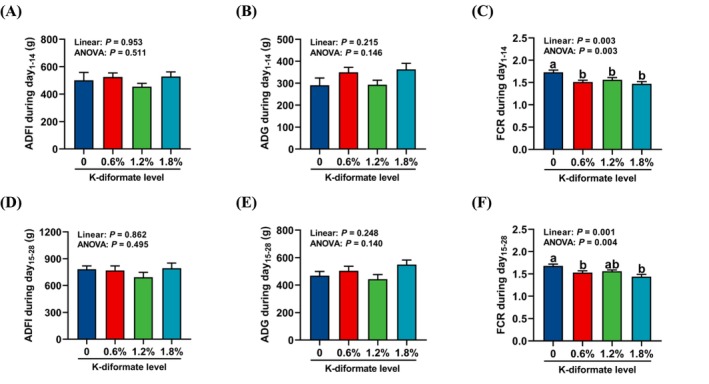
Growth performance of nursery piglets in response to dietary K‐diformate supplementation (*n* = 6 per group; mean ± SEM). (A) Average daily feed intake (ADFI) during day_1–14_. (B) Average daily gain (ADG) during day_1–14_. (C) Feed conversion ratio (FCR) during day_1–14_. (D) ADFI during day_15‐28_. (E) ADG during day_15–28_. (F) FCR during day_15–28_. Different superscript letters indicate significant differences (*p* < 0.05).

The growth performance of nursery piglets in response to dietary K‐diformate supplementation is presented in Figure 1. Neither ADFI nor ADG of piglets during days 1–14 (phase 1) and days 15–28 (phase 2) was affected by dietary treatments (*p* > 0.05). However, FCR of piglets during both phases decreased linearly with increasing K‐diformate dosage in the diet (*p* < 0.05). Importantly, piglets in the 1.8% K‐diformate group exhibited the lowest FCR, which was significantly reduced compared to the control group during both phase 1 and phase 2 (*p* < 0.05).

### 3.2 Oxidative Stress and Inflammation

**FIGURE 2:**
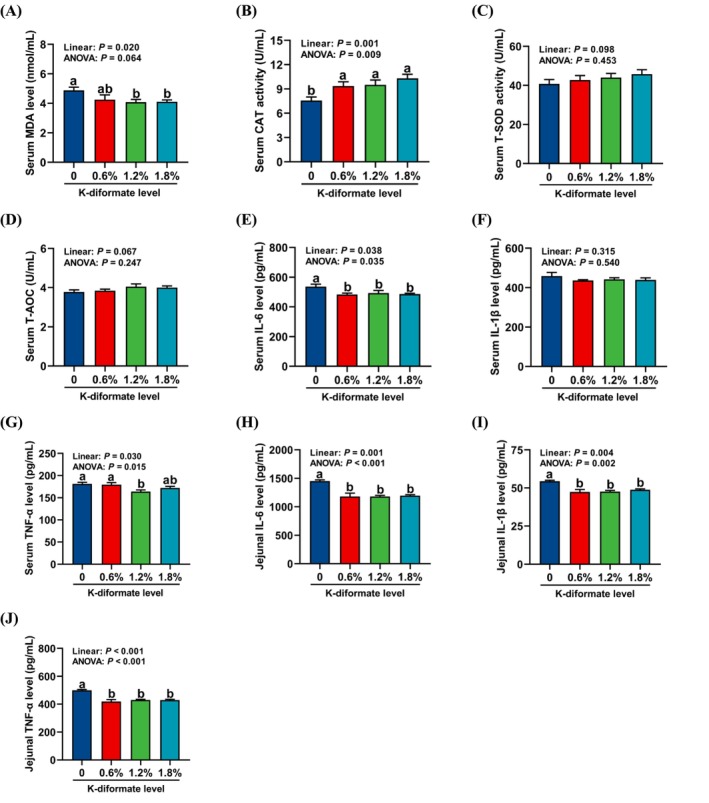
Oxidative stress and inflammation of nursery piglets in response to dietary K‐diformate supplementation (*n* = 6 per group; mean ± SEM). (A–D) Serum MDA level, CAT activity, T‐SOD activity, and T‐AOC. (E–G) Serum IL‐6, IL‐1β, and TNF‐α levels. (H–J) Jejunal IL‐6, IL‐1β, and TNF‐α levels. Different superscript letters indicate significant differences (*p* < 0.05).

As illustrated in Figure 2, T‐SOD activity and T‐AOC in the serum of piglets exhibited a linear upward trend with elevating dietary K‐diformate levels (*p* < 0.10). Additionally, serum CAT activity increased linearly, while serum MDA, IL‐6, and TNF‐α levels decreased linearly in response to rising dietary K‐diformate levels (*p* < 0.05). Regarding intestinal inflammation, jejunal IL‐6, IL‐1β, and TNF‐α levels showed a linear decline with increasing dietary K‐diformate levels (*p* < 0.05). More importantly, compared with the control group, piglets receiving 1.8% K‐diformate exhibited elevated serum CAT activity and lower serum MDA and IL‐6 levels, along with reduced jejunal IL‐6, IL‐1β, and TNF‐α levels (*p* < 0.05).

### 3.3 Intestinal Barrier Function

**FIGURE 3:**
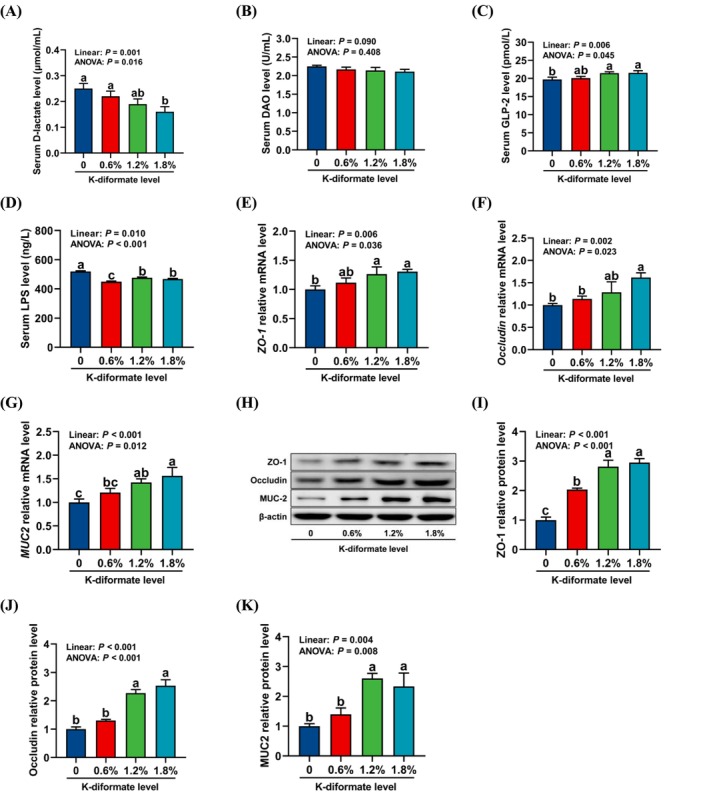
Intestinal barrier function of piglets in response to dietary K‐diformate supplementation. (A–D) Serum D‐lactate, DAO, GLP‐2, and LPS levels (*n* = 6 per group; mean ± SEM). (E–G) Jejunal relative mRNA levels of *ZO‐1*, *Occludin*, and *MUC2* (*n* = 6 per group; mean ± SEM). (H) Western blot bands. (I–K) Jejunal relative protein levels of ZO‐1, Occludin, and MUC2 (*n* = 3 per group; mean ± SEM). Different superscript letters indicate significant differences (*p* < 0.05).

As shown in Figure 3, serum D‐lactate and LPS levels (*p* < 0.05) decreased linearly, while serum DAO activities (*p* < 0.10) exhibited a trend toward linear reduction, and serum GLP‐2 levels (*p* < 0.05) increased linearly with elevating dietary K‐diformate levels. Compared to the control group, piglets in the 1.8% K‐diformate group exhibited reduced serum D‐lactate and LPS levels, along with elevated serum GLP‐2 levels (*p* < 0.05). Also, a linear increase was observed for jejunal ZO‐1, Occludin, and MUC2 with the increasing dietary K‐diformate levels, both at mRNA and protein expression levels (*p* < 0.05). And these parameters were significantly upregulated in the 1.8% K‐diformate group relative to the control group (*p* < 0.05).

### 3.4 Colonic Microbiota Diversity

**FIGURE 4:**
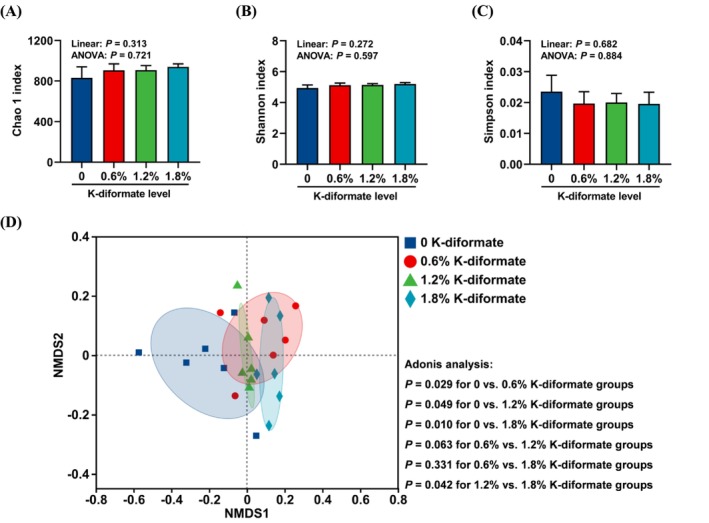
The diversity of microbiota in colon digesta of nursery piglets in response to dietary K‐diformate supplementation. (A–C) Chao 1 index, Shannon index, and Simpson index (*n* = 6 per group; mean ± SEM). (D) β‐diversity.

The diversity of microbiota in the colon digesta of nursery piglets in response to dietary K‐diformate supplementation is shown in Figure 4. The Chao 1 index, Shannon index, and Simpson index of colonic microbiota were unaffected by dietary treatments (*p* > 0.05). However, statistical differences in β‐diversity were observed between the control group and the 0.6%, 1.2%, or 1.8% K‐diformate groups (*p* < 0.05). Additionally, statistical differences in β‐diversity were found between the 1.8% K‐diformate group and the 0.6% or 1.2% K‐diformate groups (*p* < 0.05).

### 3.5 Colonic Microbiota Composition

**FIGURE 5:**
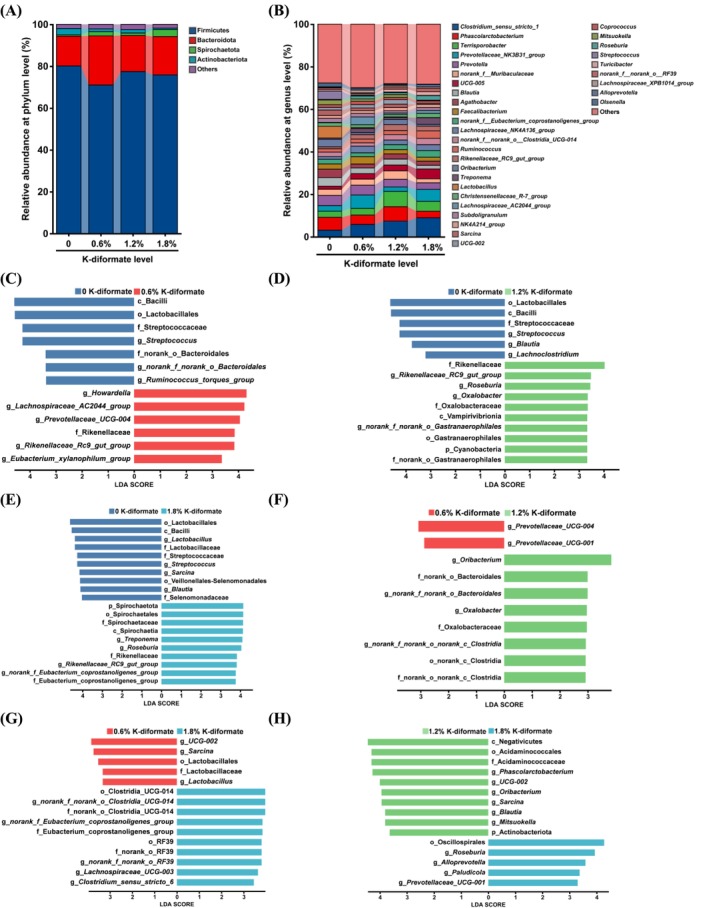
Composition of microbiota in colon digesta of nursery piglets. (A, B) Microbiota composition at phylum and genus levels (*n* = 6 per group). (C–H) LEfSe analysis of microbiota from phylum to genus levels for 0% versus 0.6% K‐diformate groups, 0% versus 1.2% K‐diformate groups, 0% versus 1.8% K‐diformate groups, 0.6% versus 1.2% K‐diformate groups, 0.6% versus 1.8% K‐diformate groups, and 1.2% versus 1.8% K‐diformate groups (LDA score > 2.0).

The composition of microbiota in colon digesta of nursery piglets in response to dietary K‐diformate supplementation is presented in Figure 5. LEfSe analysis revealed that, relative to the control group, piglets receiving 0.6% K‐diformate exhibited higher abundances of g_*Howardella*, g_*Lachnospiraceae_AC2044_group*, g_*Prevotellaceae_UCG‐004*, f_Rikenellaceae, g_*Rikenellaceae_Rc9_gut_group*, and g_*Eubacterium_xylanophilum_group*, while showing lower abundances of c_Bacilli, o_Lactobacillales, f_Streptococcaceae, g_*Streptococcus*, f_norank_o_Bacteroidales, g_*norank_f_norank_o_Bacteroidales*, and g_*Ruminococcus_torques_group* (*p* < 0.05). Compared with the control group, piglets in the 1.2% K‐diformate group had higher abundances of f_Rikenellaceae, g_*Rikenellaceae_RC9_gut_group*, g_*Roseburia*, g_*Oxalobacter*, f_Oxalobacteraceae, c_Vampirivibrionia, g_*norank_f_norank_o_Gastranaerophilales*, o_Gastranaerophilales, p_Cyanobacteria, and f_norank_o_Gastranaerophilales, while showing lower abundances of o_Lactobacillales, c_Bacilli, f_Streptococcaceae, g_*Streptococcus*, g_*Blautia*, and g_*Lachnoclostridium* (*p* < 0.05). Compared with the control group, piglets in the 1.8% K‐diformate group exhibited higher abundances of p_Spirochaetota, o_Spirochaetales, f_Spirochaetaceae, c_Spirochaetia, g_*Treponema*, g_*Roseburia*, f_Rikenellaceae, g_*Rikenellaceae_RC9_gut_group*, g_*norank_f_Eubacterium_coprostanoligenes_group*, and f_Eubacterium_coprostanoligenes_group, while showing lower abundances of o_Lactobacillales, c_Bacilli, g_*Lactobacillus*, f_Lactobacillaceae, f_Streptococcaceae, g_*Streptococcus*, g_*Sarcina*, o_Veillonellales‐Selenomonadales, g_*Blautia*, and f_Selenomonadaceae (*p* < 0.05).

When compared to the 0.6% K‐diformate group, the 1.2% K‐diformate group exhibited increased abundances of g_*Oribacterium*, f_norank_o_Bacteroidales, g_*norank_f_norank_o_Bacteroidales*, g_*Oxalobacter*, f_Oxalobacteraceae, g_*norank_f_norank_o_norank_c_Clostridia*, o_norank_c_Clostridia, and f_norank_o_norank_c_Clostridia, alongside reduced abundances of g_*Prevotellaceae_UCG‐004* and g_*Prevotellaceae_UCG‐001* (*p* < 0.05). Relative to the 0.6% K‐diformate group, the 1.8% K‐diformate group displayed higher abundances of o_Clostridia_UCG‐014, g_*norank_f_norank_o_Clostridia_UCG‐014*, f_norank_o_Clostridia_UCG‐014, g_*norank_f_Eubacterium_coprostanoligenes_group*, f_Eubacterium_coprostanoligenes_group, o_RF39, f_norank_o_RF39, g_*norank_f_norank_o_RF39*, g_*Lachnospiraceae_UCG‐003*, and g_*Clostridium_sensu_stricto_6*, with lower abundances of g_*UCG‐002*, g_*Sarcina*, o_Lactobacillales, f_Lactobacillaceae, and g_*Lactobacillus* (*p* < 0.05). Compared with the 1.2% K‐diformate group, piglets in the 1.8% K‐diformate group exhibited higher abundances of o_Oscillospirales, g_*Roseburia*, g_*Alloprevotella*, g_*Paludicola*, and g_*Prevotellaceae_UCG‐001*, alongside reduced abundances of c_Negativicutes, o_Acidaminococcales, f_Acidaminococcaceae, g_*Phascolarctobacterium*, g_*UCG‐002*, g_*Oribacterium*, g_*Sarcina*, g_*Blautia*, g_*Mitsuokella*, and p_Actinobacteriota (*p* < 0.05).

### 3.6 Predicted Microbiota Functions

**FIGURE 6:**
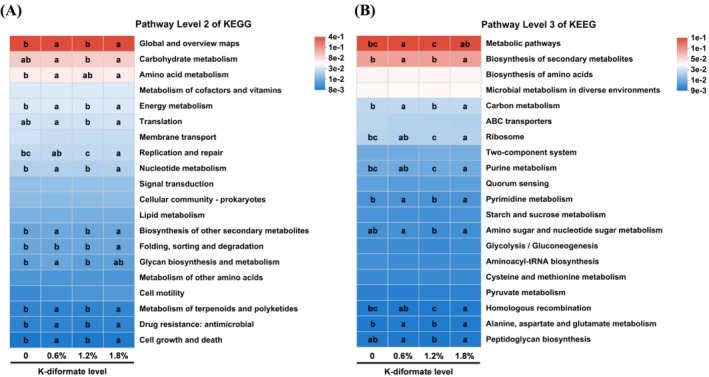
KEGG predicted functions of microbiota in colon digesta of nursery piglets in response to dietary K‐diformate supplementation (*n* = 6 per group). (A, B) Pathway level 2 and 3 of KEGG. Different superscript letters within the same row of each figure indicate significant differences (*p* < 0.05).

The KEGG‐predicted functions of microbiota in colon digesta of nursery piglets in response to dietary K‐diformate supplementation is shown in Figure 6. At pathway level 2, compared with the control group, piglets in the 1.8% K‐diformate group exhibited enhanced global and overview maps, amino acid metabolism, energy metabolism, replication and repair, nucleotide metabolism, biosynthesis of other secondary metabolites, folding, sorting and degradation, metabolism of terpenoids and polyketides, drug resistance (antimicrobial), and cell growth and death (*p* < 0.05). At pathway level 3, piglets in the 1.8% K‐diformate group exhibited elevated biosynthesis of secondary metabolites, carbon metabolism, ribosome activity, purine metabolism, pyrimidine metabolism, homologous recombination, and alanine, aspartate, and glutamate metabolism relative to the control group (*p* < 0.05).

### 3.7 Colonic Short‐Chain Fatty Acid Profile

**FIGURE 7:**
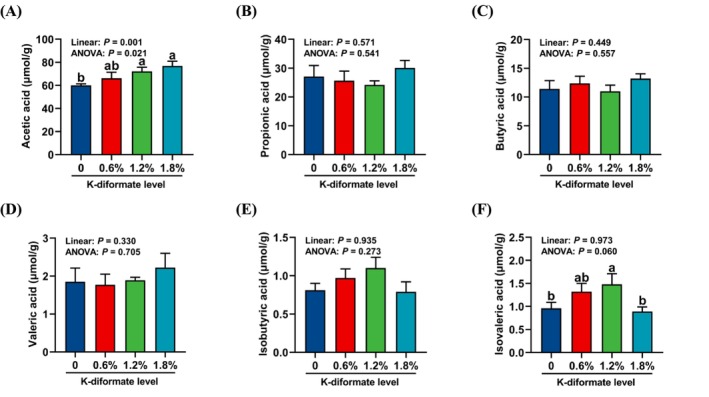
Short‐chain fatty acid profile in the colonic digesta of piglets in response to dietary K‐diformate supplementation (*n* = 6 per group; mean ± SEM). (A–F) Acetic acid, propionic acid, butyric acid, valeric acid, isobutyric acid, and isovaleric levels in the colonic digesta. Different superscript letters indicate significant differences (*p* < 0.05).

The short‐chain fatty acid profile in the colonic digesta of piglets in response to dietary K‐diformate supplementation is presented in Figure 7. The levels of propionic acid, butyric acid, valeric acid, and isobutyric acid in colonic digesta were not affected by dietary treatments (*p* > 0.05). However, the acetic acid level in colonic digesta was linearly increased with the increase of dietary K‐diformate levels (*p* < 0.05). Compared with the control group, dietary supplementation with 1.2% or 1.8% K‐diformate increased acetic acid levels in colonic digesta, while supplementation with 1.2% K‐diformate elevated isovaleric acid levels (*p* < 0.05).

### 3.8 Correlation Analysis

**FIGURE 8:**
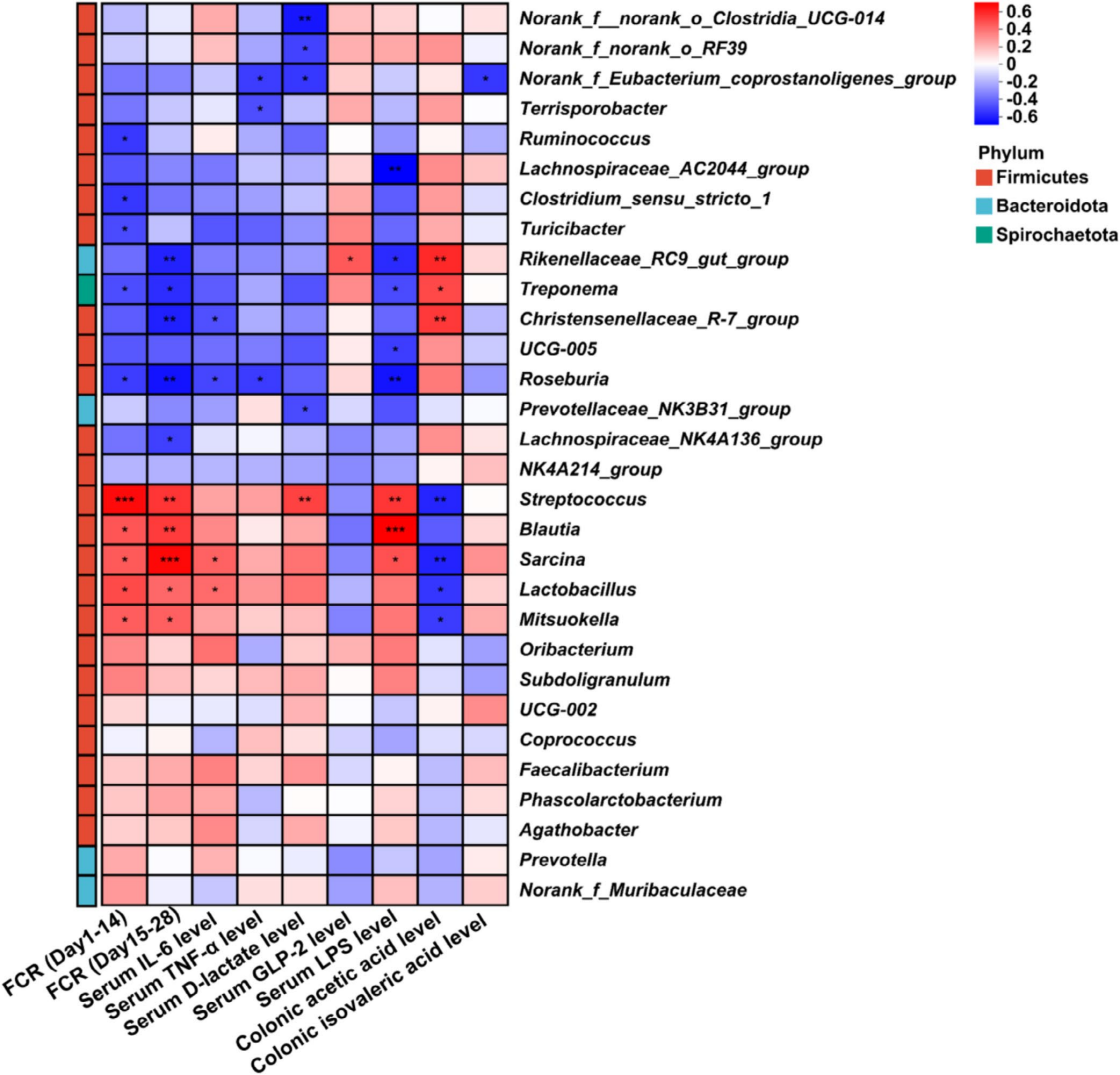
Spearman *correlation analysis of colonic microbiota at the genus level (top* 30) with FCR and serum parameters in nursery piglets. *0.01 < *p* ≤ 0.05, **0.001 < *p* ≤ 0.01, ***p ≤ 0.001.

Figure 8 displays the Spearman correlation analysis of colonic microbiota at the genus level (top 30) with FCR and serum parameters in nursery piglets. The FCR during days 1–14 and days 15–28 was negatively correlated with the abundances of *Treponema* and *Roseburia*, while positively correlated with the abundances of *Streptococcus*, *Blautia*, *Sarcina*, *Lactobacillus*, and *Mitsuokella*. Regarding these seven bacterial genera, *Treponema* abundance was negatively correlated with serum LPS levels and positively correlated with colonic acetic acid levels. *Roseburia* abundance was negatively correlated with serum IL‐6, TNF‐α, and LPS levels. *Streptococcus* abundance was negatively correlated with colonic acetic acid level and positively correlated with serum D‐lactate and LPS levels. *Blautia* abundance was positively correlated with serum LPS levels. *Sarcina* abundance was positively correlated with serum IL‐6 and LPS levels and negatively correlated with colonic acetic acid level. *Lactobacillus* abundance was positively correlated with IL‐6 levels and negatively correlated with colonic acetic acid levels. *Mitsuokella* abundance was negatively correlated with colonic acetic acid levels.

## 4 Discussion

First, we acknowledge the limitation of the small sample size (*n* = 6, one piglet per replicate). Nevertheless, this sample size is still considered sufficient and acceptable in piglet nutrition research, having met the requirements for statistical power analysis as evidenced by previous studies (Chang et al. 2023; Sun et al. 2025; Zeebone et al. 2023). In this study, results demonstrated that the FCR of piglets decreased linearly with increasing K‐diformate levels. Importantly, piglets in the 1.8% K‐diformate group exhibited the lowest FCR, which was significantly reduced compared to the control group. These findings are consistent with previous reports on FCR improvement, including studies on K‐diformate or sodium diformate supplementation in piglets (Gaffield et al. 2024; Htoo and Molares 2012; Paulicks et al. 2000), K‐diformate supplementation in broilers (Chen, Zheng, et al. 2024), and Cherry Valley ducks (Li et al. 2024). It should be noted that our study indicated a significant improvement in FCR, while ADG and ADFI were not significantly different between groups (although ADG approached a trend toward difference, with *p*‐values of 0.146 and 0.140 during weeks 1–2 and weeks 3–4, respectively). Actually, ADG and ADFI were analyzed using individual replicate values, whereas FCR was calculated as the ratio of paired ADFI/ADG values for each replicate. As a result, although the statistical difference in FCR is usually consistent with those in ADG and/or ADFI, they may occasionally differ slightly due to the ratio‐based nature of the FCR calculation. Consistent with our findings, previous piglet studies have similarly reported significant effects on FCR without significant differences in both ADG and ADFI (Chen et al. 2020; Huang et al. 2024; Jiang et al. 2015). Collectively, these studies support our findings, indicating that dietary K‐diformate supplementation, particularly at a 1.8% dosage, improved FCR in nursery piglets.

The nursery phase of piglets is characterized by oxidative stress and inflammation (Liu 2015; Yin et al. 2014). Oxidative stress is closely associated with inflammation as a pathophysiological process (Buzoglu et al. 2023; Lauridsen 2019). As such, we assessed serum oxidative stress status, followed by systemic and intestinal inflammation in piglets. The results indicated that serum oxidative stress parameters, including T‐SOD activity, T‐AOC, CAT activity, and MDA levels, exhibited a linear increase or decrease in response to elevated dietary K‐diformate levels. The results were supported by the potential antioxidant properties of K‐diformate (Sayah et al. 2024; Zhang et al. 2025). Additionally, serum IL‐6 and TNF‐α levels, as well as jejunal IL‐6, IL‐1β, and TNF‐α levels, demonstrated a linear decrease with increasing dietary K‐diformate levels. Consistent with our findings, the in vitro study by Lin et al. (2024) demonstrated that 0.125 mg/mL K‐diformate inhibited NF‐κB phosphorylation and reduced inflammation in Enterohemorrhagic *Escherichia coli* ‐challenged IPEC‐J2 cells (Lin et al. 2024). We extend this concept to living piglets and demonstrate a significant systemic relief at 1.8%, suggesting a dosage‐dependent mode of action.

Intestinal barrier dysfunction presents another challenge for nursery piglets (Tang et al. 2022). The epithelium of the small intestine functions not only as the primary site for nutrient digestion and absorption but also as a crucial protective barrier against pathogenic agents and antigens (Wijtten et al. 2011). Compromised intestinal barrier integrity or elevated permeability can lead to bacterial translocation and the systemic infiltration of allergenic substances from the intestinal lumen (Wijtten et al. 2011). We acknowledge that functional assays (e.g., FITC‐dextran permeability tests or Ussing chamber measurements) can provide direct evidence of barrier integrity. In this study, however, we used serum biomakers to indirectly assess the intestinal barrier function. Serum D‐lactate (Chen, Chen, et al. 2024), LPS (Yu et al. 2025), and GLP‐2 (Ramires et al. 2022) levels are commonly‐used biomarkers of intestinal barrier function. Compared to the control group, piglets in the 1.8% K‐diformate group exhibited reduced serum D‐lactate and LPS levels, along with elevated serum GLP‐2 levels, indicate that K‐diformate supplementation reduced paracellular and trans‐cellular leakage across intestinal epithelium. At the molecular level, piglets in the 1.8% K‐diformate group exhibited upregulated jejunal ZO‐1, Occludin, and MUC2 expression, both transcriptionally and translationally. These findings align with a prior in vitro study, where 0.125 mg/mL K‐diformate upregulated Occludin protein expression in IPEC‐J2 cells infected with enterohemorrhagic *Escherichia coli* (Lin et al. 2024). This in vitro cell culture study demonstrated that K‐diformate‐mediated upregulation of tight junction proteins contributes to the enhancement of intestinal barrier function in piglets.

The gut microbiota is greatly associated with inflammation and intestinal barrier function in piglets (Upadhaya and Kim 2022; Zeng et al. 2021). Here, we analyzed the colonic microbiota and their metabolites (short‐chain fatty acids) in colonic digesta. The results indicated that β‐diversity was significantly altered by K‐diformate supplementation, suggesting that the additive restructured the microbial community of piglets. Consistently, Lin et al. (2022) also demonstrated that dietary supplementation with K‐diformate remodeled the caecal microbiota in broilers. LEfSe analysis revealed that, relative to the control group, piglets in the 1.8% K‐diformate group at the genus level exhibited higher abundances of *Treponema*, *Roseburia*, *Rikenellaceae_RC9_gut_group*, and *norank_f_Eubacterium_coprostanoligenes_group*, while showing lower abundances of *Lactobacillus*, *Streptococcus*, *Sarcina*, and *Blautia*. Of note, an inconsistency was observed in that 1.8% K‐diformate treatment reduced the abundance of *Lactobacillus*, which is commonly considered beneficial in the pig gut. This phenomenon may be explained by the statement of Li et al. (2023), who noted that while the majority of *Lactobacillus* subspecies contribute positively to intestinal health, a fraction exhibits adverse effects. For instance, Trevisi et al. (2011) reported that dietary supplementation with *Lactobacillus rhamnosus* GG impaired the health of *Escherichia coli* F4‐infected piglets, which could partially account for the results observed in our study. Furthermore, we conducted Spearman correlation analysis to examine the relationships between colonic microbiota and their metabolic products (acetic acid and isovaleric acid levels), as well as overall host performance parameters (FCR) and systemic blood indicators (serum IL‐6, TNF‐α, D‐lactate, GLP‐2, and LPS levels). Interestingly, correlation analysis revealed that the FCR during days 1–14 and days 15–28 were negatively correlated with the abundance of *Treponema* and *Roseburia*, while positively correlated with the abundance of *Lactobacillus*, *Streptococcus*, *Sarcina*, and *Blautia*. Regarding *Treponema*, its functional complexity is considerable. Although we were unable to identify specific *Treponema* species (an acknowledged limitation of 16S sequencing compared to metagenomic approaches), a previous study has reported that some members of this genus participate in the degradation of cellulose, lignin, and resistant starch (Li et al. 2019). This may account for the increased *Treponema* abundance in gut microbiota of piglets fed diets supplemented with 1.8% K‐diformate. The results indicated that the improved FCR is partially attributed to these six bacterial genera. Interestingly, it has been reported that *Treponema* (Waugh et al. 2025), *Roseburia* (Tamanai‐Shacoori et al. 2017), *Lactobacillus* (Li et al. 2023), *Streptococcus* (Dominguez et al. 2024), *Sarcina* (Shintouo et al. 2020), and *Blautia* (Liu et al. 2021) are implicated in inflammation and/or intestinal barrier function. Interestingly, Pieszka et al. (2025) found that co‐supplementation with 1.2 kg/t potassium formate and 1.5 kg/t probiotic reduced the inflammatory response of piglets. In this regard, we further examined whether these six bacterial genera are associated with serum inflammation and intestinal barrier function biomarkers. And we found that the abundances of *Roseburia*, *Blautia*, *Sarcina*, and *Lactobacillus* were associated with inflammation, while the abundances of *Treponema*, *Roseburia*, *Streptococcus*, *Blautia*, and *Sarcina* were associated with intestinal barrier function.

Next, we utilized PICRUSt2 analysis (Liu et al. 2022) to predict enrichment of KEGG pathways. It should be noted that while this approach does not provide direct evidence of microbial metabolic activity, it enables the inference of potential metabolic functions based on 16S rRNA gene sequences. Compared with the control group, piglets in the 1.8% K‐diformate group exhibited enhanced global and overview maps, amino acid metabolism, energy metabolism, replication and repair, nucleotide metabolism, biosynthesis of other secondary metabolites, folding, sorting and degradation, metabolism of terpenoids and polyketides, drug resistance (antimicrobial), and cell growth and death. The predicted functional pathways further supported the potential association between gut microbiota modulation and the attenuation of inflammation and enhancement of intestinal barrier function in nursery piglets.

## 5 Conclusion

In conclusion, the additive effects of K‐diformate in nursery piglets exhibited dose‐dependent responses, with linear impacts on inflammation and intestinal barrier function corresponding to supplemental K‐diformate levels. In general, 1.8% K‐diformate exhibited the optimal additive effects, significantly mitigating inflammation and enhancing intestinal barrier function, which was associated with modulation of the gut microbiota.

## Author Contributions

**Yong Cheng:** data curation, investigation. **Lina Zhang:** software, investigation, methodology. **Qin Li:** methodology, investigation, software. **Mingren Qu:** supervision, conceptualization. **Songtao Zhong:** visualization, data curation. **Jun Chen:** conceptualization, writing – review and editing. **Tiande Zou:** writing – original draft, investigation, funding acquisition, conceptualization. **Li Lu:** investigation, formal analysis, validation.

## Funding

This work was supported by the Outstanding Youth Science Foundation of Jiangxi Province (20252BAC220039), National Natural Science Foundation of China (32360849), and Scientific Research Training Project of Weiyi Experimental Class of Jiangxi Agricultural University (25WY021).

## Ethics Statement

The study was approved by the Institutional Animal Care and Use Committee of Jiangxi Agricultural University (approval number: JXAULL‐20230716). All procedures adhered to institutional animal welfare guidelines, including humane endpoints (euthanasia per ethical standards) and daily health monitoring (such as diarrhea and anorexia).

## Conflicts of Interest

The authors declare no conflicts of interest.

## Data Availability

Data will be made available on request.
